# Non‐contrast based approach for liver function quantification using Bayesian‐based intravoxel incoherent motion diffusion weighted imaging: A pilot study

**DOI:** 10.1002/acm2.14178

**Published:** 2023-10-11

**Authors:** Monchai Phonlakrai, Saadallah Ramadan, John Simpson, Kate Skehan, Jonathan Goodwin, Yuvnik Trada, Jarad Martin, Swetha Sridharan, Lay Theng Gan, Sabbir Hossain Siddique, Peter Greer

**Affiliations:** ^1^ School of Health Sciences, College of Health, Medicine and Wellbeing The University of Newcastle Newcastle NSW Australia; ^2^ School of Radiological Technology Faculty of Health Science Technology Chulabhorn Royal Academy Bangkok Thailand; ^3^ HMRI Imaging Centre Hunter Medical Research Institute Newcastle NSW Australia; ^4^ College of Health, Medicine and Wellbeing The University of Newcastle Newcastle NSW Australia; ^5^ Radiation Oncology Department Calvary Mater Newcastle Newcastle NSW Australia; ^6^ School of Information and Physical Sciences, College of Engineering, Science and Environment The University of Newcastle Newcastle NSW Australia; ^7^ Faculty of Medicine and Health Sydney Medical School The University of Sydney Sydney NSW Australia; ^8^ School of Medicine and Public Health, College of Health, Medicine and Wellbeing The University of Newcastle Newcastle NSW Australia; ^9^ The Gastroenterology Department at John Hunter Hospital Newcastle NSW Australia; ^10^ Hunter New England Imaging John Hunter Hospital Newcastle NSW Australia

**Keywords:** DCE‐MRI, HCC, intravoxel incoherent motion diffusion, liver cirrhosis, liver function, T1 mapping

## Abstract

**Purpose:**

Liver cirrhosis disrupts liver function and tissue perfusion, detectable by magnetic resonance imaging (MRI). Assessing liver function at the voxel level with 13‐b value intravoxel incoherent motion diffusion‐weighted imaging (IVIM‐DWI) could aid in radiation therapy liver‐sparing treatment for patients with early impairment. This study aimed to evaluate the feasibility of IVIM‐DWI for liver function assessment and correlate it with other multiparametric (mp) MRI methods at the voxel level.

**Method:**

This study investigates the variability of apparent diffusion coefficient (ADC) derived from 13‐b value IVIM‐DWI and B1‐corrected dual flip angle (DFA) T1 mapping. Experiments were conducted in‐vitro with QIBA and NIST phantoms and in 10 healthy volunteers for IVIM‐DWI. Additionally, 12 patients underwent an mp‐MRI examination. The imaging protocol included a 13‐b value IVIM‐DWI sequence for generating IVIM parametric maps. B1‐corrected DFA T1 pulse sequence was used for generating T1 maps, and Gadoxatate low temporal resolution dynamic contrast‐enhanced (LTR‐DCE) MRI was used for generating the Hepatic extraction fraction (HEF) map. The Mann‐Whitney *U* test was employed to compare IVIM‐DWI parameters (Pure Diffusion, *D_slow_*; Pseudo diffusion, *D_fast_*; and Perfusion Fraction, *F_p_*) between the healthy volunteer and patient groups. Furthermore, in the patient group, statistical correlations were assessed at a voxel level between LTR‐DCE MRI‐derived HEF, T1 post‐Gadoxetate administration, ΔT1%, and various IVIM parameters using Pearson correlation.

**Results:**

For‐vitro measurements, the maximum coefficient of variation of the ADC and T1 parameters was 12.4% and 16.1%, respectively. The results also showed that *F_p_* and *D_fast_* were able to distinguish between healthy liver function and mild liver function impairment at the global level, with *p* = 0.002 for *F_p_* and *p* < 0.001 for *D_fast_*. Within the patient group, these parameters also exhibited a moderate correlation with HEF at the voxel level.

**Conclusion:**

Overall, the study highlighted the potential of *D_fast_* and *F_p_* for detecting liver function impairment at both global and pixel levels.

## 1 INTRODUCTION

Chronic liver disease, such as viral hepatitis and cirrhosis, is most commonly found in hepatocellular carcinoma (HCC) patients with a corresponding deleterious effect on overall liver function. The progression of the disease is typically characterized by continuously increasing hepatic fibrosis, distortion of liver tissue architecture, loss of hepatocyte density, and regenerative nodule formation.^1^ Global liver function is currently clinically evaluated and scored, with the categorical Childs Pugh Score, the most commonly used metric.^2^ However, the liver function of HCC patients with cirrhosis can be spatially heterogeneous at a sub‐regional level which is not captured by global liver function metrics.^3^ Knowledge of the spatial variation in liver function could be useful in several clinical applications including potentially liver‐sparing radiation therapy treatment where high functioning regions of the liver would be spared from the high radiation doses.^4^, ^5^, ^6^ This could result in higher retention of liver function following treatment.

Magnetic resonance imaging (MRI) is a non‐invasive imaging technique for chronic liver disease with the potential for both global and voxel‐level quantification of liver function. Gadoxetate dynamic contrast‐enhanced (DCE) MRI and T1 mapping have both been investigated to determine relative hepatocyte function globally and spatially with promising results.^7^, ^8^, ^9^ Our previous study reported the feasibility of low temporal resolution Gadoxetate DCE‐MRI (LTR‐DCE MRI) in derivation of the hepatic extraction fraction (HEF) function map in assessing spatial liver function.^10^ In addition to Gadoxetate‐based liver function, intravoxel incoherent motion diffusion‐weighted imaging (IVIM‐DWI) may facilitate quantification of both liver tissue diffusion and liver tissue perfusion without the requirement for contrast injection and the associated small risk of patient complications.

IVIM‐DWI has recently gained attention in liver function assessment. This imaging technique was initially described by Denis Le Bihan and colleagues in 1986.^11^ It is recognized as a non‐invasive MRI technique that allows for estimation of both molecular diffusion and tissue perfusion separately without gadolinium administration. This promising technique also improves the limitations of conventional DWI where the apparent diffusion coefficient (ADC) value is difficult to distinguish from the signal of blood perfusion‐driven diffusion. ADC is also less reliable where there is iron deposition in the liver.^12^ IVIM‐DWI could be particularly useful where renal function failure or allergy to Gadoxetate disodium increases the risks to patients from Gadoxetate DCE‐MRI.

Several studies have described the potential use of IVIM‐DWI in investigating global liver function and diseases. Chow et al. (2012) examined the contribution of molecular water diffusion and blood microcirculation in detecting apparent diffusion changes in early liver fibrosis in an animal model. They reported that the true diffusion (*D_slow_*) and pseudo diffusion (*D_fast_*) parameters derived from the IVIM‐DWI dataset were significantly decreased at two weeks and four weeks following carbon tetrachloride‐induced liver fibrosis, compared with that before the insult.^13^ Li et al. (2018) studied the IVIM‐DWI derived perfusion fraction (*F_p_*) as an imaging biomarker in diagnosing liver fibrosis associated with a progressive increase in connective tissue.^14^ Another study by Lefebvre and colleagues investigated the *F_p_* parameter derived from IVIM‐DWI data acquired with ten b‐values to characterize inflammation in chronic liver disease, and showed a strong correlation between *F_p_* and inflammation of the liver as measured by univariate analysis (*r*  =  − 0.70, *p*  <  0.0001).^15^ Ren et al. have compared IVIM results for 146 patients and 21 healthy volunteers using region‐of‐interest (ROI) based analysis.^16^ They found significant differences in imaging parameters between the normal control group and hepatic fibrosis group and also among liver fibrosis groups. Hectors et al.^17^ investigated the correlation between IVIM‐DWI with 16 b‐values and blood flow and perfusion parameters derived from DCE‐MRI for 25 patients using an ROI. They found significant moderate to strong negative correlations between parameters from the two methods. Murtz et al.^18^ evaluated IVIM‐DWI with three b‐values for assessing the response of HCC to transarterial loco‐regional therapies in 25 patients. Responders to therapy were found to have significant changes in parameters compared to non‐responders. Not only applying to the liver, the method has also been applied to cardiac imaging.^19^

Although there have been some investigations of the potential clinical use of IVIM in the assessment of liver function, the IVIM‐DWI map derived from the standard IVIM model using a least‐squares (LSQ) fit model at the pixel or voxel level results in very noisy estimates, particularly for the pseudo‐diffusion parameters which limits the applicability of the approach for assessing liver spatial features and heterogeneity.^20^ This problem also leads to poor reproducibility of the IVIM‐derived parameters associated with tissue perfusion.^21^ As a result, Orton et al. proposed the IVIM‐Bayesian model to improve the image quality of IVIM‐DWI in the liver by comparing results from a single slice of the liver for 20 patients with liver metastases using both LSQ and Bayesian models.^20^ The Bayesian model resulted in significantly reduced parameter estimation uncertainty and improvement in visual clarity. The method also does not require any user‐defined parameters. However, few investigations have examined the performance of IVIM‐DWI at the voxel level and utilizing the IVIM‐Bayesian model. This would seem to be a promising method to produce voxel‐level parameter maps for subsequent clinical applications.

Therefore, this study aims to assess the feasibility of 13‐b value IVIM‐DWI maps derived from the Bayesian model to evaluate liver function in early liver function impairment patients with HCC who receiving liver radiotherapy. Firstly, the reliability of ADC and T1 value measurements in phantom and in‐vivo are assessed. Discrimination of global level liver function in healthy and early liver function impairment patients was then investigated. Then, a correlation between IVIM‐DWI, HEF, and T1 mapping at a voxel level was investigated to determine the relationship between IVIM‐DWI and the more common DCE technique for determining spatial liver function.

## 2 METHODS

### 2.1 Participants

This study was ethically and scientifically reviewed and approved by the Hunter New England Human Research Ethics Committee, NSW, Australia. Twelve patients (all male, age range: 52−78 years) were prospectively enrolled in the study with informed consent. All patients were diagnosed with HCC with Childs‐Pugh A (CP‐A) disease and received liver MRI simulation scans as a part of their routine clinical workflow for liver stereotactic body radiotherapy (SBRT) treatment planning. This included routine clinical DCE‐MRI scanning performed for tumor delineation. Informed consent was collected from all patients before participating. The inclusion criteria included age over 18 years old, HCC with underlying chronic liver disease, and eligibility for MRI scanning.

A group of healthy volunteers was also recruited to participate in the same liver research project. They were included to ensure repeatability and to enable a comparison of IVIM‐DWI with the patient group. Inclusion criteria were age over 18 years and no clinical history of liver disease. Ten volunteers were recruited with a mean age of the volunteers of 32.4 ± 2.6 years. Volunteer scanning was conducted in accordance with ethical guidelines and with the explicit informed consent of participants. DCE‐MRI scanning with Gadoxetate administration was not performed for the healthy volunteers.

### 2.2 Magnetic resonance image acquisition

The liver mp‐MRI examination was undertaken on a 3T Skyra (Siemens Healthineers, Erlangen, Germany) MRI scanner with Syngo D13 software at the Radiation Oncology Department, Calvary Mater Newcastle hospital. Each patient was positioned head‐first supine on a top‐flat couch with raised arms using a T‐board as per the department's standard liver MRI simulation method. The Siemens 18‐channel body matrix phased‐array coil was positioned over the liver region. The body matrix coil was combined with the posterior spine coil to receive MRI signals.

The examination protocol began with a 13‐b value IVIM‐DWI pulse sequence. Due to the sensitivity of the echo‐planar imaging (EPI) pulse sequence to magnetic field inhomogeneity, external magnetic field shimming was enabled for the liver region before IVIM‐DWI scanning. The 13‐b value IVIM‐DWI image data was then acquired with a free‐breathing approach in the axial plane.

Next, axial 3D dual flip angle (DFA) T1‐weighted imaging was then obtained before contrast injection with a single breath‐hold utilized for each volume acquisition. B_1_‐field inhomogeneity can cause image contrast variation and affect the measurement of T1 values. Therefore, the inbuilt B1‐field inhomogeneity correction sequence (turbo fast low angle shot T1‐weighted) provided by the vendor was utilized to acquire a correction map for the liver volume prior to the above images.

Then, a non‐contrast axial 3D Dixon spoiled gradient recalled (SPGR) T1‐weighted pulse sequence was acquired after the completion of the pre‐contrast B_1_‐corrected T1 mapping acquisition to be used as a baseline liver volume image prior to contrast injection. This was followed by LTR‐DCE MRI image data, acquired during multiple breath‐holds using an axial 3D SPGR T1‐weighted Dixon pulse sequence. The LTR‐DCE MRI method was described in our previous study.^10^

Finally, post‐Gadoxetate administration DFA T1 mapping and hepatobiliary phase images were obtained at 900 s and 1200 s after injection, respectively. We acknowledge the consensus clinical protocol for gadoxetate DCE‐MRI in hepatocellular carcinomas, which recommends scanning the hepatobiliary phase (HBP) at 20 min after injection. This timing allows for the peak accumulation of gadoxetate in hepatocyte cells before a significant decrease occurs due to liver excretion processes. In accordance with this protocol, we performed T1 mapping for 900 s and conducted LTR DCE‐MRI, which lasted until 1200 s after injection.^22^ The imaging parameters of the mp‐MRI imaging protocol are detailed in Table 1.

**TABLE 1:** Imaging parameter summary of the mp‐MRI acquisition protocol.

	mp‐MRI		
Parameter	13‐b value IVIM‐DWI	3D B1‐corrected DFA‐T1 mapping	LTR‐DCE MRI (Dixon)
FOV (cm^2^)	40 × 32	40 × 32	35 × 42
Interpolated matrix size	340 × 218	448 × 360	202 × 320
TR (ms)	3000	5.08	4.29
TE (ms)	66	2.29	1.27/2.5 (TE2)
TA	5 min	15 s	15 s
Slice thickness (mm)	5	5	3
Voxel size (mm^2^)	1.176 × 1.176	0.893 × 0.893	1.312 × 1.312
FA	90^0^	3^0^ and 15^0^	9^0^
PAT factor	2	3	3
b‐value	0,10,20,30,40,50,60,70,80, 100, 200, 400, and 800 s/mm^2^	n/a	n/a
number of excitations	10–50 s/mm^2^ = 1 60−400 s/mm^2^ = 2 800 s/mm^2^ = 3	1	1
Image acquisition strategy	Free breathing	Single breath‐hold	Multiple breath‐holds

Abbreviations: DFA, dual flip angle; FOV, field‐of‐view; IVIM‐DWI, intravoxel incoherent motion; PAT, parallel acquisition technology; TA, time of acquisition; TE, time of echo; TR, rime of repetition.

The IVIM‐DWI data were obtained from ten healthy volunteers, who underwent imaging protocols and techniques identical to those used in patients, as previously described.

### 2.3 IVIM‐DWI map derivation

The IVIM‐DWI datasets were imported into Matlab Version 2019b (The Mathworks, Patick, PA, USA) to perform image post‐processing and generate IVIM‐DWI parametric maps. A median filter with a region size of 3 × 3 pixels was applied to each slice of the IVIM‐DWI dataset to reduce image noise. A binary image volume of the liver was created in 3D slicer software (http://www.slicer.org) using manual liver contour delineation and imported into Matlab for liver volume segmentation. The matrix size of each axial slice of the IVIM‐DWI dataset was down‐sampled from 340 × 218 to 128 × 128. Then, the Bayesian model was fit to each voxel independently to reduce computation time using Equation (1). Finally, the data was upsampled back to the original matrix size. This aims to generate IVIM parametric maps (*D_slow_*, pure molecular‐based diffusion coefficient; *D_fast_*, pseudo‐diffusion coefficient; and *F_P_*, pseudo‐diffusion perfusion fraction) using a MATLAB script developed by Oscar Jalnefjord.^23^

(1) $$\begin{array}{ccc} \frac{S_{n}}{S_{0}} & & =\left[F_{P}\times e^{\left(-b_{n}\cdot D_{fast}\right)}\right]\\ & & +\left[\left(1-F_{p}\right)\times e^{\left(-b_{n}\cdot D_{slow}\right)}\right]+\xi n \end{array}$$

where *S_n_* is the signal intensity of the diffusion image measured at b‐value *b_n_, S_0_* is the IVIM signal of non‐diffusion weighting (*b_0_*), *F_p_* is the perfusion fraction influenced by pseudo‐diffusion*, D_fast_* is the pseudo‐diffusion rate constant or blood perfusion‐driven diffusion, *D_slow_* is water molecule diffusion, and *ξ_n_* is an error term based on Gaussian with variance (*σ^2^_y_*)

**FIGURE 1:**
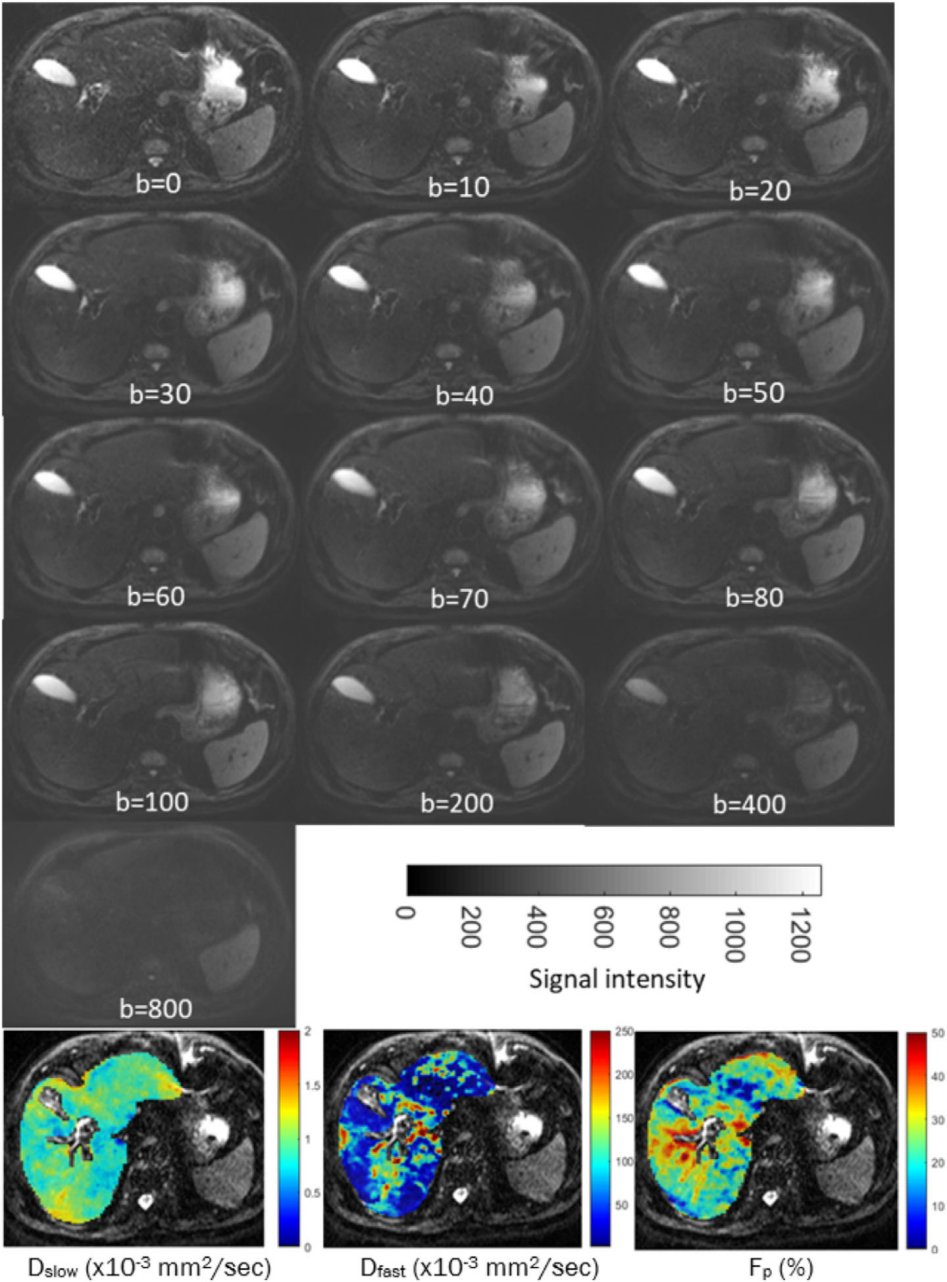
Example of 13‐b value IVIM‐DWI images with corresponding b‐value (s/mm^2^) and Bayesian‐based IVIM‐DWI derived parametric maps (*D_slow_*, *D_fast_*, and *F_p_*).

The IVIM‐Bayesian model was initially described in a study by Orton et al.,^20^ who proposed this model to improve the limitations of the constrained least‐squares (LSQ) fitting, which suffers from noisy images and limited radiological interpretability. A comprehensive summary of the Bayesian model is outlined further in Supplementary material 1. An advantage of the Bayesian model over the least square fitting model, most commonly used to estimate IVIM parameters, is that it offers a more flexible, robust, and transparent approach to modeling data compared to least square models.^19^, ^20^ Examples of IVIM‐DWI images and parametric maps are shown in Figure 1.

### 2.4 Dual flip angle (DFA) T1 map and HEF map derivation

After Gadoxetate administration, the contrast agent is taken up by the hepatocytes, which changes the T1 relaxation time of the healthy liver tissue. By performing T1 mapping before and after Gadoxetate administration, it is possible to measure the change in T1 relaxation time of the liver tissue, which provides an indirect measure of liver function. Dual flip angle (DFA) T1 mapping is a method used in MRI to quantify liver function by measuring the T1 relaxation time of liver tissue. T1 relaxation time is a measure of the time it takes for hydrogen nuclei to return to their equilibrium state after being excited by a magnetic pulse. By using two different flip angles (three and 15 degrees) during the MRI acquisition, T1 mapping can produce a map of the T1 values across the liver tissue, which can provide information on liver function. In this study, voxelwise DFA T1 maps were generated in‐line on the MRI workstation with the Syngo platform (software version, MR D13) using non‐linear least‐square curve fitting. DFA T1 maps were obtained for both pre (denoted as T1_pre_) and 15‐min post Gadoxetate administration (denoted as T1_post_), allowing for calculation of the differences in T1 relaxation time at a voxel level represented by delta T1 (ΔT1). The maximum percentage of T1 changes indicates healthy liver tissue due to shortening T1 relaxation induced by accumulated Gadoxetate in hepatocytes. The percent change of T1 can be calculated using the following Equation (2) also used in a study by Haimerl et al. (2017).^24^

(2) $$\begin{array}{ccc} & & Percentage\;T1\;reduction\left(\Delta T1\%\right)\\ & & \;=\frac{\left(T1_{pre}-T1_{post}\right)}{T1_{pre}}\times 100 \end{array}$$

**FIGURE 2:**
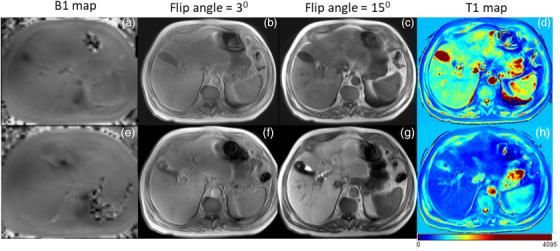
Upper panel illustrates B1 map (a), T1WI contrast with flip angle of 3 degrees (b) and 15 degrees (c), and pre Gadoxetate administration T1 mapping (d) compared to 15‐min post Gadoxetate administration B1 map (e), T1WI contrast with flip angle of 3 degrees (f) and 15 degrees (g), and T1 mapping (h) in lower panel.

An example of a T1‐weighted contrast image obtained with DFA of three degrees and 15 degrees along with corresponding T1 maps is shown in Figure 2. These were obtained from an HCC patient with CP‐A disease.

In addition, hepatic extraction fraction (HEF) maps were generated from the LTR‐DCE MRI image data using the voxelwise truncated singular value decomposition‐deconvolution analysis (TSVD‐DA) method similar to that described by Nilsson et al.^8^ and implemented in a previous study by the authors.^10^

### 2.5 Repeatability measurements in phantom and with healthy volunteers

#### 2.5.1 Phantoms

The variability of two acquisition protocols, 13‐b value IVIM‐DWI and B1‐corrected DFA T1 mapping, was assessed using the QIBA diffusion phantom (standard model 128) and NIST/ISMRM system phantom. To obtain diffusion images, the QIBA diffusion phantom was imaged three times consecutively over four sessions, resulting in a total of 12 phantom image datasets. For the DFA T1 variability measurement, the NIST phantom was imaged in two sessions. The apparent diffusion coefficient (ADC) values and T1 values across all tubes and datasets were measured using an ROI approach. The average ADC value and T1 values were compared to the reference value and the percentage difference (%ΔADC and %ΔT1), calculated across all tubes. Both intra‐ and inter‐session coefficient of variations (intra‐session CV and inter‐session CV) were measured. Further details can be found in Supplementary material 2.

#### 2.5.2 Healthy volunteers

The scan‐rescan repeatability of identical 13‐b value IVIM acquisition protocol in the ten healthy liver volunteers was assessed. Two MRI scan sessions were performed, with an average interval of 37.6 days (±34.3 days, SD) without fasting. *D_slow_*, *D_fast_*, and *F_p_* maps were generated for the entire liver volume, and the average and SD of all parameters were computed for within‐subject coefficient of variation (_W_CV) analysis.

### 2.6 Data analysis

IVIM‐derived parameters were compared between the healthy volunteer group and the HCC patient group with CP‐A level disease. Global *D_slow_*, *D_fast_*_,_ and *F_p_* values were averaged from all voxels within the segmented liver volume and compared between the two groups. In addition, all subjects were classified as healthy = 0 or disease = 1 to assess the diagnostic performance of the IVIM‐derived parameters in discriminating liver dysfunction from a healthy liver.

To examine the relationship between the parameter values at a voxel level from the different MRI techniques, rigid image co‐registration across HEF maps, T1 maps, and IVIM parametric maps was performed using 3D slicer software before exporting to MATLAB for quantification. Then, a single 2D ROI (1585 ± 370 voxels, mean ± 1SD) was manually drawn over segment VIII of the liver HEF map on one slice that exhibited a high level of registration agreement to create a binary mask image before applying it to the T1_pre_, T1_post_, *D_slow_*, *D_fast_*, and *F_p_* maps to extract parametric values. T1_pre_ and T1_post_ values were used to calculate the percentage of T1 decrease (∆T1%). Voxel values of HEF, T1_post_, ∆T1%, *D_slow_*, *D_fast_*, and *F_p_* were utilized to evaluate voxel wise correlation through linear correlation for individuals within the patient group.

### 2.7 Statistics

SPSS Statistics Version 27.0 was used to analyze the patient data. IVIM‐DWI values were expressed in mean, median, max, min, and SD. The nonparametric Mann‐Whitney test was used to compare the global *D_slow_*, *F_p_*, and *D_fast_* between the mild liver function impairment patient and healthy volunteer groups. Furthermore, a receiver operating characteristic or ROC curve was used to assess the diagnostic performance of the *D_slow_*, *F_p_*, *D_fast_* parameters in discrimination of patient and healthy volunteer groups. The area under the ROC curve was reported as a diagnostic performance index. Internal validation was performed using Bootstrapping (number of samples = 1000, confidence level = 95%). A p‐value less than 0.05 was considered statistically significant in all tests.

SPSS software (IBM® SPSS® Statistics 27) was utilized to test normality using the Kolmogorov–Smirnov test. The data were non‐normally distributed (*p* < 0.001). However, as the sample size was larger than 100 voxels, the violation of the normality assumption would not be expected to cause significant issues.^25^, ^26^, ^27^ To evaluate the voxel wise correlation among MRI‐derived parameters in the 12 patients, the Pearson correlation coefficient was used. The range of values for the Pearson correlation coefficient, *r*, is between +1 and −1. When *r* = 0, it suggests that there is no connection between the two variables. If r is greater than 0, it indicates a positive association, meaning that as one variable increases, so does the other variable. On the other hand, if r is less than 0, it suggests a negative association, which means that as one variable increases, the other variable decreases. a correlation coefficient of r between 0.1 and 0.3 (or −0.1 to −0.3) suggests a small strength of association, whereas a coefficient between 0.3 and 0.5 (or −0.3 to −0.5) implies a medium strength of association. A coefficient between 0.5 and 1.0 (or −0.5 to −1.0) indicates a large strength of association. The larger the absolute value of the correlation coefficient (*r*), the stronger the relationship between the two variables. The Fisher *Z* transformation was employed to calculate the average correlation coefficient (*r*) across patients, which involved transforming the *r*‐value to Fisher's *Z* value and then calculating an averaged *r*.

## 3 RESULTS

### 3.1 Repeatability measurements in phantom and with healthy volunteers

The phantom repeatability measurements and results are given in the Supplementary material 2 (Table S1). Measured ADC values for the IVIM‐DWI acquisition protocol had less than a 2% difference from known values, except for the highest value which was 7.7%. Intra‐session variability was 7.3% and inter‐session variability was 12.4% according to the coefficient of variation.

For the B1‐corrected DFA T1 mapping acquisition protocol, the percentage difference between measured and reference T1 relaxation times for the phantom ranged from 0.9% to 13.0%. The intra‐session variability was within 3.2% except for the two highest concentration vials which had a maximum of 13.2%. The inter‐session variability was within 4.1% except for the same two vials which had a maximum of 16.1% (Table S2)

The repeatability of the measurements in the healthy volunteer cohort resulted in an average intra‐subject variability of 3.0% (0.3%–9.3%), 10.4% (1.9%–25.2%), and 8.6% (2.6%–13.5%) for *D_slow_*, *D_fast_*, and *F_p_*, respectively (Table S3)

### 3.2 Comparison of global IVIM‐derived parameters between healthy volunteers and patients

The quantitative results are given in Table 2. The median values (±SD) of the IVIM‐DWI derived *D_slow_*, *F_p_*, and *D_fast_* parameters in healthy volunteers were 1.02 ± 0.15 × 10^−3^ mm^2^/s, 29.2% ± 5.1%, and 73.5 ± 25.5 × 10^−3^ mm^2^/s, respectively. In comparison, the values in the patient group with mild liver function impairment were 0.96 ± 0.13 × 10^−3^ mm^2^/s, 21.6 ± 4.0%, and 36.4 ± 14.2 × 10^−3^ mm^2^/s respectively. A significant difference in the *F_p_* and *D_fast_* parameters between the two groups was observed (*p* = 0.001 and *p* < 0.0001, respectively). In contrast, there was no significant difference between the two groups for the *D_slow_* parameter.

**TABLE 2:** Descriptive statistics of IVIM‐DWI results for the healthy volunteers compared to mild liver function impairment patients. The data represents global‐level averages from all liver voxels.

	Healthy volunteers (*n* = 10)				Cirrhotic patients (*n* = 12)				
Parameter	Median	Min	Max	SD	Median	Min	Max	SD	*P*‐value
*D_slow_*	1.02	0.87	1.27	0.15	0.96	0.72	1.16	0.13	=0.075
*F_p_*	29.2	22.3	39.6	5.1	21.6	13.6	28.1	4.0	=0.002*
*D_fast_*	73.5	49.0	133.6	25.5	36.4	17.6	60.2	14.2	<0.001*

Abbreviations: D_fast_, Pseudo diffusivity (x10^−3^ mm^2^/s); *D_slow_*, water molecule diffusivity (x10^−3^ mm^2^/s); *F_p_*, perfusion fraction (%).

*Significant difference at *p*‐value < 0.005 using Mann–Whitney *U* test.

**FIGURE 3:**
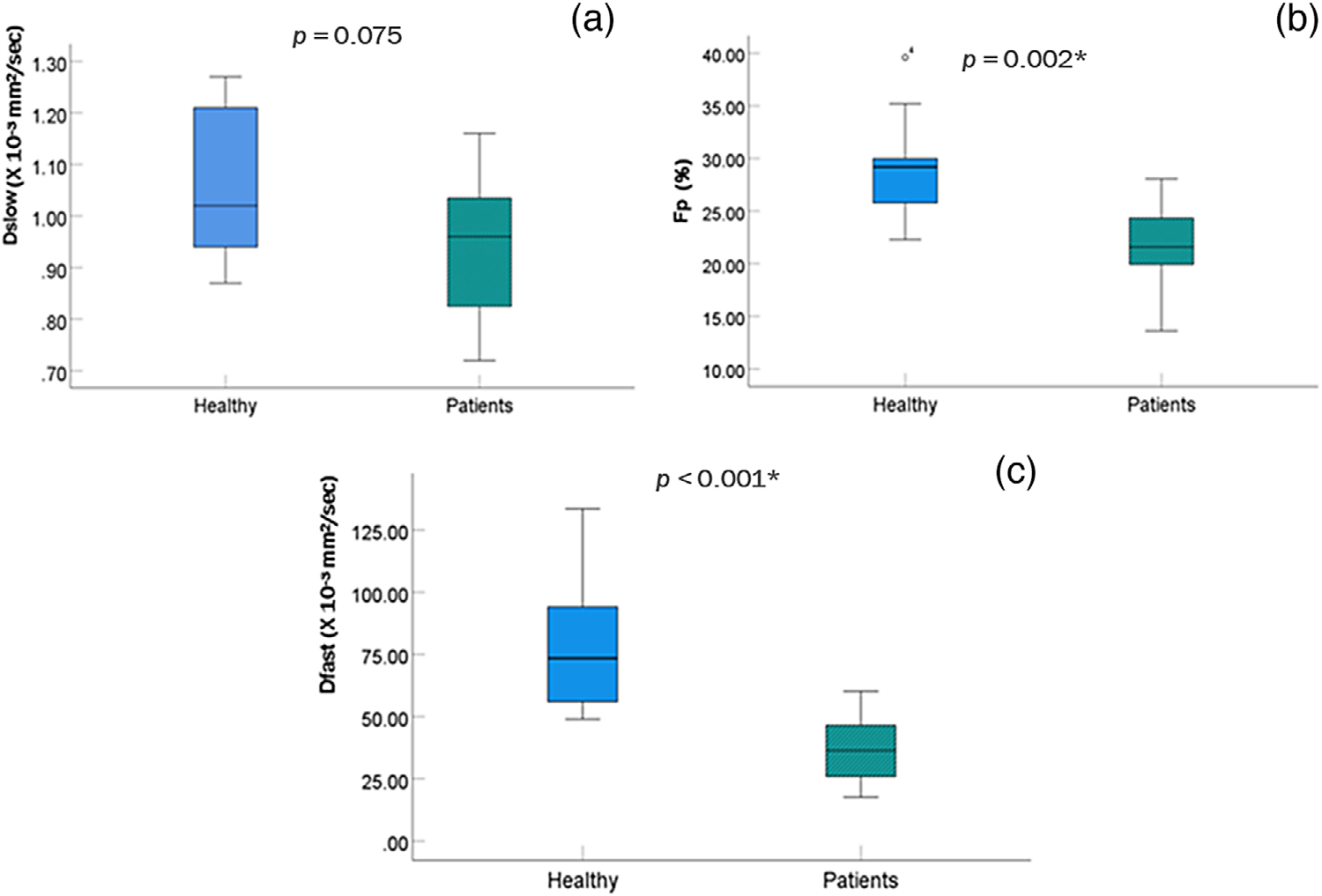
Boxplots compare *D_slow_* (a), *F_p_* (b), and *D_fast_* (c) between healthy volunteers and patients. *D_fast_* and *F_p_* were significantly different between healthy and CP‐A patients with HCC using the Mann–Whitney *U* test. * Significant difference at *p*‐value < 0.05.

Figure 3 shows the quantitative value comparison of *D_slow_* (a), *F_p_* (b), and *D_fast_* (c) between the two groups. The blue boxplot shows the healthy volunteer results, and the green is the patient group. The results show that the parameters significantly decreased (*p* < 0.05) in the liver mild function impairment patients except for *D_slow_* (*p* = 0.075) which did not meet statistical significance.

### 3.3 Diagnostic performance of 13‐b value IVIM‐DWI derived parameters in discriminating healthy liver from CP‐A disease

**FIGURE 4:**
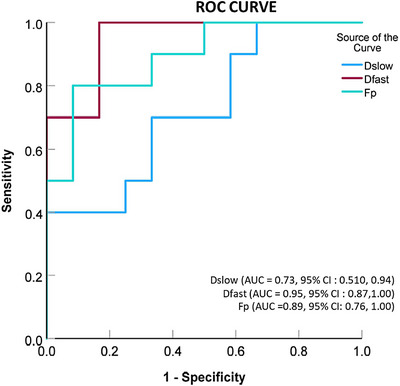
The ROC curve analysis demonstrates *D_slow_*, *F_p_*, and *D_fast_* diagnostic performance in classifying healthy liver from HCC patients with mild liver function impairment disease.

Figure 4 shows the ROC curve for the two groups to evaluate the diagnostic performance of IVIM‐derived parameters in discriminating global liver function. The area under the ROC curve is calculated for three parameters—*D_slow_*, *F_p_*, and *D_fast_*. The area under the ROC curve for *F_p_* and *D_fast_* is higher than that for *D_slow_*, indicating that they are better parameters for discriminating global liver function.

The optimal cutoff values for *F_p_*, and *D_fast_* for discriminating the healthy liver from the CP‐A liver were found to be 25.3% (sensitivity = 0.80, specificity = 0.917) and 58.5 × 10^−3^ mm^2^/s (sensitivity = 0.700, specificity = 0.917), respectively.

Figure S1 in the supplementary material provides an example comparing *D_slow_*, *D_fast_*, and *F_p_* maps of a healthy volunteer and a patient.

### 3.4 Voxel wise correlation across MRI‐derived parameters within the patient group

The average dice similarity score between binary masks of all parametric maps, which estimates the quality of the image registration index, was 0.85 ± 0.02. Table 3 shows the voxel wise correlation coefficients or *r*‐values with 95% CI between HEF, *D_slow_*, *D_fast_*, *F_p_*, T1_post_, and ∆T1% for the 12 patients. For the HEF parameter, the *r*‐value between HEF and other parameters varied across patients, from low to moderate positive and negative correlations.

**TABLE 3:** Statistical summary of voxel wise mp‐MRI correlation and 95% confidence intervals within the patient group.

	Correlation coefficient (*r*) [95% Confidence Intervals]											
PT.	HEF‐*D_slow_*	HEF‐*D_fast_*	HEF‐*F_p_*	HEF‐T1_post_	HEF‐∆T1	*D_slow_*‐T1_post_	*D_slow_*‐∆T1	*D_fast_*‐*F_p_*	*D_fast_*‐T1_post_	*D_fast_*‐∆T1	*F_p_*‐T1_post_	*F_p_*‐∆T1
P1	−0.45 [−0.49, −0.40]	0.27 [0.21, 0.32]	0.40 [0.35, 0.45]	−0.72 [−0.74, −0.69]	0.55 [0.50, 0.59]	0.44 [0.40, 0.49]	−0.63 [−0.67, −0.60]	0.27 [0.21, 0.32]	−0.51 [−0.55, −0.47]	0.11 [0.06, 0.17]	−0.41 [−0.46, −0.36]	0.50 [0.45, 0.54]
P2	−0.34 [−0.39, −0.29]	0.29 [0.24, 0.34]	0.31 [0.26, 0.37]	−0.52 [−0.56, −0.48]	0.19 [0.14, 0.25]	0.36 [0.31, 0.41]	−0.23 [−0.28, −0.18]	0.79 [0.77, 0.81]	−0.11 [−0.16, −0.05]	−0.05 [−0.11, 0.01]	−0.17 [−0.22, −0.11]	−0.02 [−0.07, −0.04]
P3	−0.20 [−0.26, −0.15]	0.24 [0.19, 0.29]	0.34 [0.29, 0.39]	0.41 [0.37, 0.46]	0.45 [0.41, 0.49]	−0.78 [−0.80, −0.75]	−0.44 [−0.48, −0.39]	0.38 [0.34, 0.43]	0.25 [0.20, 0.30]	0.24 [0.19, 0.29]	0.87 [0.86, 0.89]	0.57 [0.53, 0.60]
P4	−0.62 [−0.68, −0.55]	0.44 [0.35, 0.52]	0.25 [0.14, 0.34]	−0.03 [−0.13, 0.08]	0.22 [0.12, 0.32]	0.43 [0.34, 0.51]	−0.58 [−0.64, −0.50]	0.69 [0.63, 0.74]	−0.20 [−0.30, −0.10]	0.27 [0.17, 0.37]	−0.19 [−0.28, −0.08]	0.26 [0.16, 0.36]
P5	−0.50 [−0.58, −0.43]	0.63 [0.56, 0.68]	0.48 [0.39, 0.55]	−0.30 [−0.39, −0.21]	0.60 [0.53, 0.66]	0.50 [0.42, 0.57]	−0.70 [−0.75, −0.64]	0.33 [0.24, 0.42]	−0.28 [−0.37, −0.19]	0.37 [0.28, 0.45]	−0.33 [−0.42, −0.24]	0.56 [0.49, 0.62]
P6	−0.64 [−0.72, −0.55]	0.15 [−0.00, 0.29]	0.38 [0.24, 0.50]	−0.13 [−0.27, 0.03]	0.36 [0.22, 0.48]	0.29 [0.15, 0.43]	−0.48 [−0.59, −0.36]	0.46 [0.34, 0.57]	−0.37 [−0.49, −0.23]	0.42 [0.28, 0.53]	−0.46 [−0.57, −0.34]	0.45 [0.32, 0.56]
P7	−0.22 [−0.28, −0.16]	0.21 [0.16, 0.27]	0.42 [0.37, 0.47]	−0.68 [−0.71, −0.65]	0.46 [0.41, 0.51]	0.08 [0.02, 0.14]	−0.21 [−0.26, −0.15]	0.24 [0.18, 0.30]	−0.19 [−0.25, −0.13]	0.16 [0.10, 0.21]	−0.33 [−0.40, −0.28]	0.16 [0.10, 0.22]
P8	−0.69 [−0.72, −0.67]	0.54 [0.50, 0.57]	0.74 [0.72, 0.76]	−0.64 [−0.67, −0.61]	0.74 [0.72, 0.76]	0.59 [0.56, 0.62]	−0.72 [−0.74, −0.70]	0.71 [0.67, 0.74]	−0.23 [−0.27, −0.18]	0.20 [0.15, 0.24]	−0.41 [−0.45, −0.37]	0.41 [0.37, 0.45]
P9	0.02 [−0.09, 0.13]	0.12 [0.01, 0.23]	0.34 [0.24, 0.43]	−0.53 [−0.60, −0.44]	0.45 [0.36, 0.53]	−0.04 [−0.15, 0.07]	−0.35 [−0.44, −0.25]	0.64 [0.57, 0.70]	−0.19 [−0.30, −0.08]	−0.22 [−0.32, −0.11]	−0.40 [−0.49, −0.31]	−0.17 [−0.28, −0.07]
P10	0.38 [0.33, 0.44]	0.42 [0.37, 0.47]	0.16 [0.10, 0.22]	−0.00 [−0.07, 0.06]	0.24 [0.18, 0.30]	−0.31 [−0.37, −0.25]	0.48 [0.43, 0.52]	0.50 [0.44, 0.54]	0.13 [0.06, 0.19]	0.15 [0.09, 0.21]	0.00 [−0.07, 0.06]	0.09 [0.03, 0.15]
P11	−0.64 [−0.68, −0.59]	0.28 [0.22, 0.34]	0.53 [0.48, 0.58]	−0.74 [−0.77, −0.71]	0.21 [0.14, 0.28]	0.74 [0.71, 0.77]	−0.43 [−0.48, −0.37]	0.50 [0.45, 0.55]	−0.40 [−0.45, −0.34]	0.11 [0.04, 0.18]	−0.56 [−0.61, −0.51]	0.19 [0.13, 0.26]
P12	−0.13 [−0.17, −0.09]	0.33 [0.30, 0.37]	0.34 [0.30, 0.37]	−0.24 [−0.28, −0.20]	0.47 [−0.48, −0.37]	0.04 [0.00, 0.08]	−0.17 [−0.21, −0.13]	0.53 [0.50, 0.56]	−0.42 [−0.46, −0.39]	0.50 [0.04, 0.18]	0.06 [−0.10, −0.02]	0.26 [0.23, 0.30]
$\bar{z}$	−0.39	0.35	0.43	−0.41	0.46	0.21	−0.42	0.58	−0.22	0.20	−0.18	0.26
$\bar{r}$	−0.37	0.34	0.45	−0.39	0.43	0.21	−0.4	0.52	−0.22	0.2	−0.18	0.25
Min	−0.69	0.12	0.16	−0.74	0.19	−0.78	−0.72	0.24	−0.51	−0.22	−0.56	−0.17
Max	0.38	0.63	0.74	0.41	0.74	0.74	0.48	0.79	0.25	0.42	0.87	0.57

Abbreviations: $\bar{r}$, the average value of the correlation coefficient; $\bar{z}$, the average value of Fisher's *Z*; PT, patient.

HEF had a moderate negative correlation with *D_slow_* and T1_post_ parameters with the average correlation coefficients for the patients being ($\bar{r}$ = −0.37; range, −0.69, 0.38; and $\bar{r}$ = −0.39; range, −0.74, 0.41, respectively). However, no significant correlation was found in a patient (P9) for HEF and *D_slow_* and three patients (P4, P6, & P10) for HEF and T1_post_ . In contrast, HEF had a moderate positive correlation with *D_fast_* ($\bar{r}$ = 0.34; range, 0.12, 0.63), *F_p_* ($\bar{r}$ = 0.45; range, 0.16, 0.74), and ∆T1% ($\bar{r}$ = 0.43; range, 0.19, 0.74). No significant correlation was found in a patient (P6) for HEF and *D_fast_*.

*D_slow_* exhibited a weak positive correlation with T1_post_ ($\bar{r}$ = 0.21; range, −0.78, 0.74), however, no significant correlation was found in a patient (P9). In addition, *D_slow_* had a moderate negative correlation with ∆T1 ($\bar{r}$ = −0.40; range, −0.72, 0.48). All patients showed a significant correlation between *D_slow_* and ∆T1.

*D_fast_* was correlated with *F_p_* ($\bar{r}$ = 0.52; range, 0.24, 0.79), but it had a weak correlation with T1_post_ and ∆T1% ($\bar{r}$ = −0.22; range, −0.51, 0.25; and $\bar{r}$ = 0.20; range, −0.22, 0.42), respectively. No significant correlation was found in one patient (P2) for *D_fast_* and ∆T1%.

**FIGURE 5:**
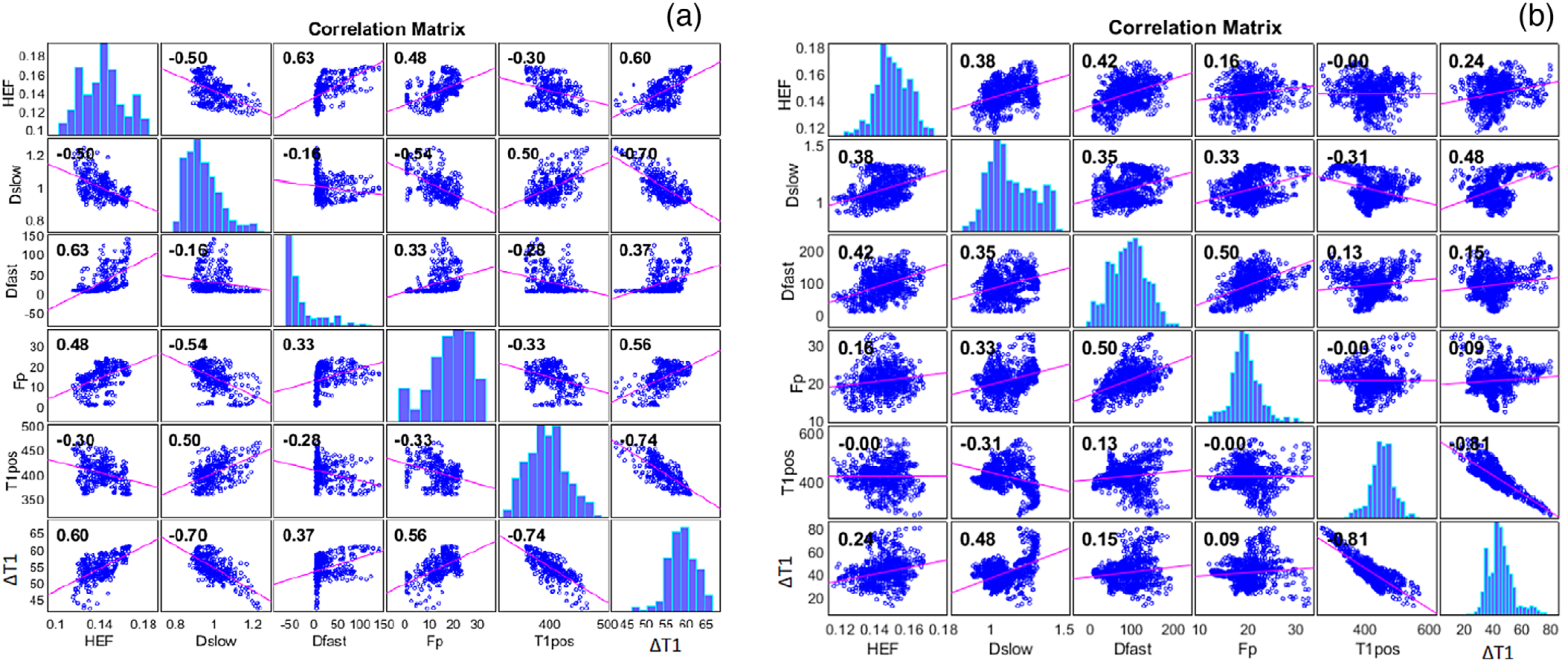
Example of the multiparametric MRI voxelwise linear correlation results for a CP‐A patient P5 (a) given a good result and P10 (b) given a weak correlation.

*F_p_* had weak relationships with T1_post_ and ∆T1% ($\bar{r}$ = −0.17; range, −0.56, 0.87 and $\bar{r}$ = 0.25; range, −0.17, 0.57), respectively. No significant correlation was found in P10 for *F_p_* and T1_post_ and in P2 for *F_p_* and ∆T1%. Figure 5 shows an example of the voxel‐based correlation analysis among mp‐MRI derived parameters, with patient P5 (a) showing a strong correlation and P10 (b) a weak correlation. The details of *p*‐values table are provided in the Supplementary material 2 (Table S4).

Figure S2 in the Supplementary material 3 gives an example of liver segmented MRI images of arterial phase (a) and hepatobiliary phase (b), LTR‐DCE MRI derived HEF maps (c), Bayesian‐based 13‐b value IVIM‐DWI derived parametric maps *D_slow_* (d), *D_fast_* (e), and *F_p_* (f), as well as B1‐corrected T1 maps, including T1_pre_ (g) and T1_post_ (h), in an HCC patient with CP‐A disease. The asterisk indicates the tumor region.

## 4 DISCUSSION

This paper deployed multiparametric MRI for liver function quantification for multiple liver tissue features, including tissue diffusivity, liver tissue blood perfusion, and hepatocellular function. DCE‐MRI–derived HEF, T1_post_, and ∆T1% parametric maps are contrast‐based functional parameters and were used to determine relative hepatocyte function. On the other hand, IVIM‐DWI derived *D_slow_*, *D_fast_*_,_ and *F_p_* are non‐contrast based functional parameters. Therefore, they were used here to estimate liver tissue diffusivity (*D_slow_*) and liver tissue blood perfusion (*D_fast_* and *F_p_*).

The 13‐b value IVIM‐DWI and B1 corrected DFA T1 mapping acquisition protocols were assessed for accuracy and repeatability using phantom testing. For IVIM‐DWI, the agreement to reference values and repeatability of the ADC value was assessed. The 50% PVP concentration in the diffusion phantom showed a greater difference to the reference value than the other concentrations. This was possible due to the high PVP concentration tube containing air pockets that can cause outlier ADC values in a high PVP concentration phantom as described by Wagner et al. (2017).^28^ According to the measurement in this present study, the intra‐ and inter‐session repeatability were regarded as reasonable with the maximum CV of 7.3% and 12.5%, respectively.

For the T1 mapping the measured T1 difference to reference values was a maximum of 13.6%. This problem can potentially be ameliorated by using the difference in T1 values pre‐ and post‐ Gadoxetate injection (∆T1%). However, this is still affected by intra‐session repeatability which was in general low but for some concentrations was of a similar magnitude to above. For the repeatability measurements, both intra‐ and inter‐session variability increased with increasing NiCI_2_ concentration. The increased T1 variation with the increased NiCl_2_ concentration is probably due to increased magnetic susceptibility in higher NiCl_2_ concentrations.^29^

Keenan et al. (2016)^30^ investigated the variability of inversion recovery and variable flip angle T1 across centers and MRI vendors using the NIST phantom. From their measurements, T1 variability at 3T was up to 26% compared to this current study, which was 16.1%. The T1 variation trends in the current study were comparable to that measured from vendor A in the Keenan study using inversion recovery T1 mapping, which also showed increased T1 variation in high NiCl_2_ concentrations. Additionally, a recent study by Keenan et al.’s study (2021)^31^ stated that the largest variations and bias in T1 measurement were observed for VFA measurement at 3T. They suggested that the error in VFA measurement might be due to imperfect B1 fields and associated nonregular slice profiles, as VFA measurements are highly susceptible to this source of error. The flip angle is directly proportional to B1 field strength, and the relative error in T1 is approximately twice that of the relative error in the flip angle. Our study's results are consistent with their findings, which also demonstrated significant variation in T1 values obtained from VFA T1 mapping sequence, particularly in high NiCl_2_ concentration tubes.

For in‐vivo repeatability testing of the 13‐b value IVIM‐DWI acquisition protocol on ten healthy volunteers, the *D_slow_* parameter had the lowest variability followed by *F_p_*. In comparison, the *D_fast_* parameter had a higher variability with an average wCV of 10.4% and a range of 1.9%–25.2%. The largest uncertainty in IVIM is attributed to fitting error resulting in higher variability in the *D_fast_* parameter.^32^ However, the variability of *D_fast_* in this investigation was lower than that reported by Cieszanowski et al.,^33^ deploying a free‐breathing 10‐b value IVIM acquisition protocol at 3T acquired with 3−6 signal acquisitions (CV ranged from 50% to 80%). These results are therefore generally encouraging for the use of global parameter values from IVIM DWI imaging for quantifying liver function.

Despite higher variability than the other parameters, the global *D_fast_* parameter could distinguish between healthy volunteers and mild liver function impairment patients. Global *D_fast_* and *F_p_* were significantly lower in patients compared to the healthy liver function group, although only mild liver function impairment patients were recruited. *D_slow_* tended to be lower in patients compared with the healthy volunteers, but no significant difference was found in this study owing to small sample size. The decrease of *F_p_* and *D_fast_* in the cirrhotic patients in this study is consistent with a previous study by Lefebvre and colleagues, reporting a negative correlation between *F_p_* and increased liver dysfunction grade (*r*  = −0.70, *p* < 0.0001).^15^ In addition, *F_p_* and *D_fast_* had a high diagnostic performance in the discrimination of healthy and mild liver function impairment when assessed with ROC analysis.

Besides global liver function analysis, this study also investigated the feasibility of voxel wise analysis of IVIM maps derived from the Bayesian model. Using this method, noise can be reduced to improve voxel‐based comparison. The results from voxel‐based analysis illustrated that HEF was significantly correlated with liver tissue perfusion‐related parameters (*D_fast_* and *F_p_*) and hepatocyte function‐associated parameters (∆T1%) in almost all patients, however, the correlations were not strong. A relationship between HEF and the ∆T1% value was also seen in a study by Haimerl et al., who reported that ∆T1% in normal liver function was significantly higher than that in cirrhotic patients at a global level.^34^ The positive correlations from this study suggest that liver voxels with higher function would be associated with higher HEF, *F_p_*, *D_fast_*, and ∆T1% than voxels with reduced liver function.

HEF was negatively correlated with the *D_slow_* parameter associated with tissue diffusivity in most patients; however, some patients exhibited positive correlations. This might be caused by uncertainties in image registration. Additionally, the observed weak and heterogeneous correlation between HEF and *D_slow_* values could also stem from subtle alterations in tissue architecture within this patient cohort. The *D_slow_* parameter was not found to clearly distinguish liver dysfunction in this study where normal volunteers were compared to CP‐A dysfunction patients. A study by Koinuma et al. reported a negative relationship between ADC value and grade of liver function impairment. Wang et al. (2018) also reported that liver tissue stiffness due to fibrosis positively correlated with CP class.^35^

HEF was also negatively correlated with the T1_post_ parameter. HEF values decrease for cirrhotic patients, whereas higher T1 values would be expected due to lower Gadoxetate uptake. The T1 value of normal hepatocyte function voxels decrease 15‐min post‐Gadoxetate administration due to Gadoxetate‐induced shortening of the T1 relaxation time.^36^ Our results show a positive correlation between HEF and ΔT1%, which aligns with the findings in Haimerl's study (2017)^24^ showing that ΔT1% (denoted as rrT1% in their study) tended to decrease in more severe liver fibrosis. This correlation is reasonable because a normal liver function typically exhibits high HEF values and high ΔT1%. Moreover, as *D_fast_* and *F_p_* are related to liver tissue perfusion, *D_fast_* was positively correlated with the *F_p_* parameter, as would be expected.

There are several limitations to this study. First, in‐vivo repeatability of T1 values was not investigated in this study, and this study was not able to acquire mp‐MRI data in the normal volunteers due to the Gadoxetate administration required for DCE‐MRI and T1 mapping. Thus, only the IVIM‐DWI data were acquired in healthy volunteers and compared with the patient group. Secondly, the number of patients was small, mainly due to the COVID‐19 pandemic, resulting in limited patient recruitment. This could reflect the low diagnostic performance of the *D_slow_* parameter in distinguishing between normal and cirrhotic liver. Thirdly, the study did not validate IVIM‐DWI with different grades of liver dysfunction as all patients were diagnosed with the same liver dysfunction grade of CP‐A disease. However, the results showed the capability of global *D_fast_* and *F_p_* parameters in discriminating the early stages of liver dysfunction from healthy function.

Another limitation is that the IVIM‐DWI scans were acquired with free breathing while the LTR DCE‐MRI was acquired in breath‐hold. This is necessary due to the long imaging time of the sequence. This, combined with the poor anatomical visualization for the DWI imaging technique results in image registration uncertainties when registering to the DCE‐MRI scans. Furthermore, interpolating voxel sizes to match the reference HEF map, particularly in the slice direction could potentially misalign IVIM map voxels with the reference voxels. To minimize registration uncertainty, only a single slice of the registered data was used for the parameter comparisons. Moreover, motion of the patient during the IVIM‐DWI scan can also reduce the image quality. This will affect the voxel wise comparison results and may explain the lack of strong correlations. Therefore, an IVIM‐DWI acquisition protocol incorporating a novel motion‐insensitive technique would be suggested to improve image quality for further study. This will also be beneficial for clinical implementation of this imaging technique if combined with DCE‐MRI to determine spatial liver function.

## 5 CONCLUSION

This pilot study investigated the feasibility of 13‐b value IVIM‐DWI and B1‐corrected dual flip angle T1 mapping acquisition protocols in quantifying liver function. The IVIM‐derived *F_p_* and *D_fast_* parameters could distinguish healthy liver function and mild liver function impairment at the global level and showed correlation with the Gadoxetate LTR‐DCE MRI‐based HEF parameter at the voxel level within the patient group despite the small number of patients and differences in patient motion and position. The results from this study suggest that the addition of the IVIM‐DWI acquisition and *D_fast_* and *F_p_* parameters could potentially be useful in assessing liver tissue perfusion however greater patient numbers are required, and methods to consolidate patient positioning. Furthermore, results from this work also found a positive relationship between ∆T1% and HEF parameters, which are associated with hepatocyte function at the voxel level. However, further study is required to determine whether T1 mapping would improve liver function quantification or could be an alternative method.

## AUTHOR CONTRIBUTIONS

Peter Greer, Saadallah Ramadan, and John Simpson supervised the work and contributed to the guarantors of integrity of entire study, study concepts/study design, approval of final version of submitted manuscript, and manuscript drafting or manuscript revision for important intellectual content. Monchai Phonlakrai performed literature research, the experiments, and data/statistical analysis. Kate Skehan performed data acquisition and manuscript drafting and manuscript revision for important intellectual content. Jonathan Goodwin, Yuvnik Trada, Jarad Martin, Swetha Sridharan, Lay Theng Gan, and Sabbir Hossain Siddique contributed to the manuscript drafting and manuscript revision for important intellectual content. All authors provided critical feedback and helped shape the research, analysis, and manuscript.

## CONFLICT OF INTEREST STATEMENT

The authors declare no conflicts of interest.

## Supporting information

Supporting Information

Supporting Information

Supporting Information
