# Supplementary material for: Non‐contrast based approach for liver function quantification using Bayesian‐based intravoxel incoherent motion diffusion weighted imaging: A pilot study
Source: J Appl Clin Med Phys. 2023 Oct 11;24(11):e14178. doi: 10.1002/acm2.14178 (PMC10647975; doi:10.1002/acm2.14178)
Supplement: Supplementary file 1 — Supporting Information [file ACM2-24-e14178-s002.docx]

**Supplementary materials 1**

**1. Bayesian-based IVIM-DWI Model**

1.1 IVIM Signal Model and Data Likelihood

The Bayes theorem determines the probability of an event is based on previous information (prior) about the conditions associated with the event. In this study, the Bayes theorem was used to estimate the probability of IVIM-derived parametric values, particularly in low signal-voxel events. The Gaussian distribution of the signal histogram of the non-diffusion (S_0_) map within the liver volume is defined as a prior distribution using mean and variance. This inference method [160] is summarized as hierarchical Bayesian modelling combined with the IVIM model as:


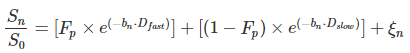


(1)

where ***S_n_*** is the signal intensity of diffusion image measured at b-value of ***b_n_, S_0_*** is the IVIM signals of non-diffusion weighting (b_0_), ***F_p_*** is the perfusion fraction influenced by pseudo-diffusion*,* ***D_fast_*** is the pseudo-diffusion rate constant or blood perfusion-driven diffusion, ***D_slow_*** is water molecule diffusion, and ***ξn*** is an error term based on Gaussian with variance (***σ^2^_y_***) expressed as


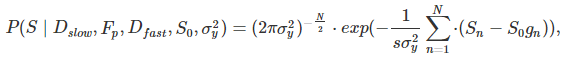

 (2)

Where :

*S =[S1,S2,....,Sn]^T^*g*_n_* = [F_p_⋅exp (−b⋅D_fast_)] +[(1−F_p_)⋅exp(−b⋅D_slow_) ]
*N* =number of b−values

In general, parameters ( $S_{0},\sigma_{y}^{2}$), which are still required to complete the model, can be expressed in the Bayesian method by applying an integrated likelihood function using a conjugate Normal-Inverse-Gamma prior distribution as given by


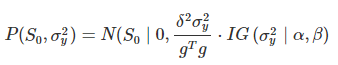


(3)

where *g* = [*g* _1_,  *g* _2_, …*g* *_N_* ]*^T^*, *N*(·) is a Gaussian distribution, and *IG* (·) is an Inverse-Gamma distribution. Then, the integration over the domain of definition is applied as


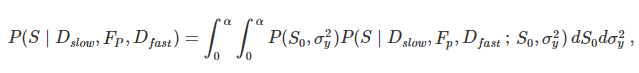


(4)


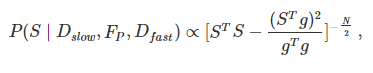
In Equation 4, the upper limit value of the variance of the normal distribution of S_0_ map is defined as *δ* → ∞ . In addition, shape and scale parameters or minimum value of the Inverse Gamma distribution of the $S_{0}$ map are defined as *α*, *β* → 0. This aims to encode the complete lack of prior information to ensure that the estimated $S_{0}$will be influenced by the real data rather than the prior. As a consequence, the marginalized likelihood function is represented by

(5)

1.2. Bayesian Shrinkage Prior

This step will estimate the value of IVIM parameters once $S_{0}$ map is known. To this end, a hierarchical prior structure is deployed to know a prior distribution over transformed ***D_slow_***, ***F_p_*,** and ***D_fast_*** parameters before applying Bayesian inference or multivariate Gaussian on these parameters. The constraints of the parameters are defined by giving that 0 < ***F_p_*** < 1, 0 < ***D_slow_***, and 0 < ***D_fast_*** . Then, the transformed IVIM parameters within the domain of definition are mapped onto the field of real numbers as given by

d=log(D_slow_)

f=logit(F_p_)=log(F_p_)−log(1−F_p_)

d^∗^=log(D_fast_)

Prior distribution describes the heterogeneity across voxels within the segmented liver volume and models correlations between the parameters via the covariance matrix *Σ_μ_*  as


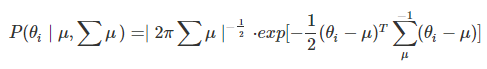
 (6)

Where :

*θ_i_*  = [d_i_, f_i_, d^∗^_i_] of voxel i

*μ* = [μ_d_,μ_f_,μ_d∗_] is the mean of segment liver volume

*∑μ* is a 3 x 3 symmetric covariance matrix.

The prior specification is then completed with hyper-prior  μ and Σ_μ_ using Jeffrey’s prior [182] as


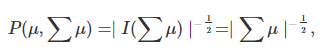


(7)

The prior in Equation 7 utilises the Fisher Information, ***I(∑μ)****,* to estimate IVIM parameter value for good signal voxels determined as a high probability. On the other hand, in case of poor signal voxels on the S_0_ map, the parameter covariance matrix, ***∑μ*** , will be considered. Therefore, a “shrinking” of parameter estimation using the mean of the distribution will be performed. As a result, the posterior distribution of IVIM parameters can be estimated.

1.3. Posterior Inference

Based on Bayes theorem, the joint posterior distribution for a segmented liver volume containing M voxels is given by


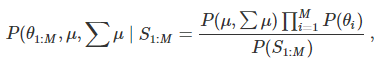
 (8)


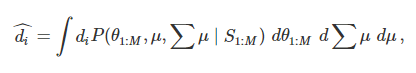
where *M* is the total number of voxels. As the parameter independent data evidence, *P(S_1:M_),* is not required for the inference procedure, expected values of IVIM parameters under the posterior in Equation 7 are thus calculated by

(9)

and similarly for *f_i_* and $d_{i}^{*}$ , when summary statistics for segment liver volume are of interest, an estimator of μ and its elements is needed and given by


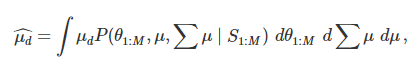
 (10)


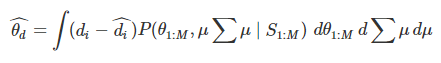
for $\mu_{d}$. In addition, $\mu_{f}$ and $\mu_{d^{*}}$ are estimated using the same equation. To give heterogeneity or correlation measures over the segmented liver volume, the *∑μ* of each parameter can also be estimated using the following equation.

(11)

1.4. Markov chain Monte Carlo (MCMC) implementation

The integration in Equation 9 requires a heavy computational task over a very large number of variables. The MCMC-based numerical approach is therefore used to generate a sequence of samples (N_s_) from a Markov chain to approximate IVIM parameters [160], for example, $d$ parameter


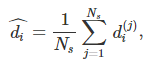


(12)

where $d_{i}^{(j)}$ is d parameter with sample *j* of voxel *i.*
