# Supplementary material for: Non‐contrast based approach for liver function quantification using Bayesian‐based intravoxel incoherent motion diffusion weighted imaging: A pilot study
Source: J Appl Clin Med Phys. 2023 Oct 11;24(11):e14178. doi: 10.1002/acm2.14178 (PMC10647975; doi:10.1002/acm2.14178)
Supplement: Supplementary file 2 — Supporting Information [file ACM2-24-e14178-s003.docx]

**Supplementary Tables**

| **Table S1.** Summary of ADC values measured from all the QIBA phantom vial tubes using the 13-b value IVIM-DWI acquisition protocol. | | | | |  |
| --- | --- | --- | --- | --- | --- |
| Vial plate number | PVP conc.  % | Ref. ADC   (10^-3^ mm^2^/s) | Measured ADC   (10^-3^ mm^2^/s) | ∆ADC% |  |
| 1-3 | 0 | 1.12 | 1.11 ± 0.36 | -0.9 |  |
| 4-5 | 10 | 0.84 | 0.83 ± 0.15 | -1.2 |  |
| 6-7 | 20 | 0.61 | 0.62 ± 0.12 | 1.6 |  |
| 8-9 | 30 | 0.40 | 0.40 ± 0.07 | 0.0 |  |
| 10-11 | 40 | 0.25 | 0.25 ± 0.04 | 0.0 |  |
| 12-13 | 50 | 0.13 | 0.12 ± 0.02 | -7.7 |  |
| **Abbreviations**: PVP, polymer polyvinylpyrrolidone; Ref, Reference; ADC, apparent diffusion coefficient; conc., concentration | | | | | |

| Table S2. Average T1 measured from two sessions of MRI scans and percentage T1 difference for each concentration | | | | | | |
| --- | --- | --- | --- | --- | --- | --- |
| NiCl_2_ rod No. | Conc.(mM) | Ref. T1 (ms) | B1-corrected DFA T1 acquisition protocol | | | %T1 difference |
|  |  |  | T1 of 1^st^ session (ms) | T1 of 2^nd^ session (ms) | Average |  |
| 1 | 0.30 | 1989.00 | 2005.52 | 2009.28 | 2007.40 | 0.9 |
| 2 | 0.62 | 1454.00 | 1377.88 | 1389.64 | 1383.76 | -4.8 |
| 3 | 1.07 | 984.10 | 999.76 | 1004.08 | 1001.92 | 1.8 |
| 4 | 1.72 | 706.00 | 770.22 | 768.56 | 769.39 | 9.0 |
| 5 | 2.62 | 496.70 | 533.90 | 535.28 | 534.59 | 7.6 |
| 6 | 3.91 | 351.50 | 323.76 | 320.04 | 321.90 | -8.4 |
| 7 | 5.73 | 247.13 | 214.26 | 212.94 | 213.60 | -13.6 |
| 8 | 8.30 | 175.30 | 159.04 | 159.00 | 159.02 | -9.3 |
| 9 | 11.94 | 125.90 | 123.10 | 123.32 | 123.21 | -2.1 |
| 10 | 17.07 | 89.00 | 88.10 | 88.52 | 88.31 | -0.8 |
| 11 | 24.33 | 62.70 | 56.58 | 56.14 | 56.36 | -10.1 |
| 12 | 34.59 | 44.53 | 50.60 | 50.08 | 50.34 | 13.0 |
| 13 | 49.12 | 30.84 | 34.50 | 31.42 | 32.96 | 6.9 |
| 14 | 69.68 | 21.72 | 22.64 | 23.52 | 23.08 | 6.3 |
| Abbreviation: Conc, concentration; mM, millimole; Ref, Referent; % T1 difference, percentage of the mean difference between reference T1 and measured T1; NiCl2, NiCl2 - Nickel(II) chloride. | | | | | | |

| **Table S3*.*** Statistical summary of D_slow_, D_fast_ and F_p_ quantifications in ten healthy volunteers | | | | | | | | | |
| --- | --- | --- | --- | --- | --- | --- | --- | --- | --- |
| Volunteer | D_slow__S1 | D_slow__S2 | wCV_Dslow_ | D_fast__S1 | D_fast__S2 | wCV_Dfast_ | F_p__S1 | F_p__S2 | wCV_Fp_ |
| 1 | 1.27 | 1.29 | 1.0% | 68.41 | 61.66 | 7.3% | 29.3 | 34.9 | 12.2% |
| 2 | 1.21 | 1.23 | 1.1% | 54.39 | 62.64 | 10.0% | 22.3 | 24.4 | 6.2% |
| 3 | 0.93 | 0.91 | 1.3% | 133.55 | 99.89 | 20.4% | 35.2 | 29.1 | 13.3% |
| 4 | 1.03 | 0.96 | 5.3% | 99.92 | 88.12 | 8.9% | 39.6 | 42.1 | 4.4% |
| 5 | 1.00 | 1.00 | 0.5% | 49.00 | 70.27 | 25.2% | 24.5 | 22.2 | 7.0% |
| 6 | 0.87 | 0.86 | 0.3% | 73.28 | 77.00 | 3.5% | 29.1 | 34.9 | 12.9% |
| 7 | 1.01 | 1.02 | 0.8% | 94.14 | 82.88 | 9.0% | 30.0 | 24.7 | 13.5% |
| 8 | 1.08 | 1.05 | 2.2% | 73.89 | 88.45 | 12.7% | 29.5 | 27.2 | 5.6% |
| 9 | 1.27 | 1.12 | 9.3% | 73.73 | 78.79 | 4.7% | 26.5 | 25.5 | 2.6% |
| 10 | 0.94 | 1.05 | 8.0% | 56.03 | 57.53 | 1.9% | 25.8 | 28.8 | 7.9% |
| Average | 1.06 | 1.05 | 3.0% | 77.63 | 76.72 | 10.4% | 29.17 | 29.39 | 8.6% |
| Median | 1.02 | 1.04 | 1.2% | 73.50 | 77.90 | 8.9% | 29.22 | 28.01 | 7.4% |
| Min | 0.87 | 0.86 | 0.3% | 49.00 | 57.53 | 1.9% | 22.32 | 22.20 | 2.6% |
| Max | 1.27 | 1.29 | 9.3% | 133.55 | 99.89 | 25.2% | 39.60 | 42.13 | 13.5% |
| SD | 0.15 | 0.13 | 3.3% | 25.51 | 13.67 | 7.4% | 5.09 | 6.16 | 4.1% |
| **Abbreviations**: S1 and S2, first and second sessions; D_slow_, water molecule diffusion (x10^-3^ mm^2^/s); D_fast_ , Pseudo diffusion (x10^-3^ mm^2^/s); F_p_, Perfusion Fraction (%); wCV, within-subject Coefficient of variation | | | | | | | | | |

| **Table S4.**  The p-value of voxelwise mp-MRI parameter correlations | | | | | | | | | | | | | | |
| --- | --- | --- | --- | --- | --- | --- | --- | --- | --- | --- | --- | --- | --- | --- |
| PT | p-value | | | | | | | | | | | | | |
|  | HEF-D_slow_ | HEF-D_fast_ | HEF-F_p_ | HEF-T1_post_ | HEF-∆T1 | D_slow_-T1_post_ | | | D_slow_-∆T1 | D_fast_-F_p_ | D_fast_-T1_post_ | D_fast_-∆T1 | F_p_-T1_post_ | F_p_-∆T1 |
| P1 | <0.001 | <0.001 | <0.001 | <0.001 | <0.001 | <0.001 | <0.001 | | | <0.001 | <0.001 | =0.000 | <0.001 | <0.001 |
| P2 | <0.001 | <0.001 | <0.001 | <0.001 | <0.001 | <0.001 | | <0.001 | | <0.001 | <0.001 | =0.095 | <0.001 | =0.558 |
| P3 | <0.001 | <0.001 | <0.001 | <0.001 | <0.001 | <0.001 | | <0.001 | | <0.001 | <0.001 | <0.001 | <0.001 | <0.001 |
| P4 | <0.001 | <0.001 | <0.001 | =0.587 | <0.001 | <0.001 | | <0.001 | | <0.001 | =0.000 | <0.001 | =0.001 | <0.001 |
| P5 | <0.001 | <0.001 | <0.001 | <0.001 | <0.001 | <0.001 | | <0.001 | | <0.001 | <0.001 | <0.001 | <0.001 | <0.001 |
| P6 | <0.001 | =0.055 | <0.001 | =0.102 | <0.001 | <0.001 | | <0.001 | | <0.001 | <0.001 | <0.001 | <0.001 | <0.001 |
| P7 | <0.001 | <0.001 | <0.001 | <0.001 | <0.001 | <0.011 | | <0.001 | | <0.001 | <0.001 | <0.001 | <0.001 | <0.001 |
| P8 | <0.001 | <0.001 | <0.001 | <0.001 | <0.001 | <0.001 | | <0.001 | | <0.001 | <0.001 | <0.001 | <0.001 | <0.001 |
| P9 | =0.67 | =0.031 | <0.001 | <0.001 | <0.001 | =0.454 | | <0.001 | | <0.001 | =0.001 | =0.000 | <0.001 | =0.002 |
| P10 | <0.001 | <0.001 | <0.001 | =0.960 | <0.001 | <0.001 | | <0.001 | | <0.001 | <0.001 | <0.001 | =0.920 | = 0.006 |
| P11 | <0.001 | <0.001 | <0.001 | <0.001 | <0.001 | <0.001 | | <0.001 | | <0.001 | <0.001 | =0.002 | <0.001 | <0.001 |
| P12 | <0.001 | <0.001 | <0.001 | <0.001 | <0.001 | =0.040 | | <0.001 | | <0.001 | <0.001 | <0.001 | =0.004 | <0.001 |
| **Abbreviations:** PT, Patient; <0.1 and < 0.05, significant correlation at p-value < 0.01 and 0.05 using Pearson correlation coefficient | | | | | | | | | | | | | | |
