# Supplementary material for: Non‐contrast based approach for liver function quantification using Bayesian‐based intravoxel incoherent motion diffusion weighted imaging: A pilot study
Source: J Appl Clin Med Phys. 2023 Oct 11;24(11):e14178. doi: 10.1002/acm2.14178 (PMC10647975; doi:10.1002/acm2.14178)
Supplement: Supplementary file 3 — Supporting Information [file ACM2-24-e14178-s001.docx]

**Supplementary Figures**

**
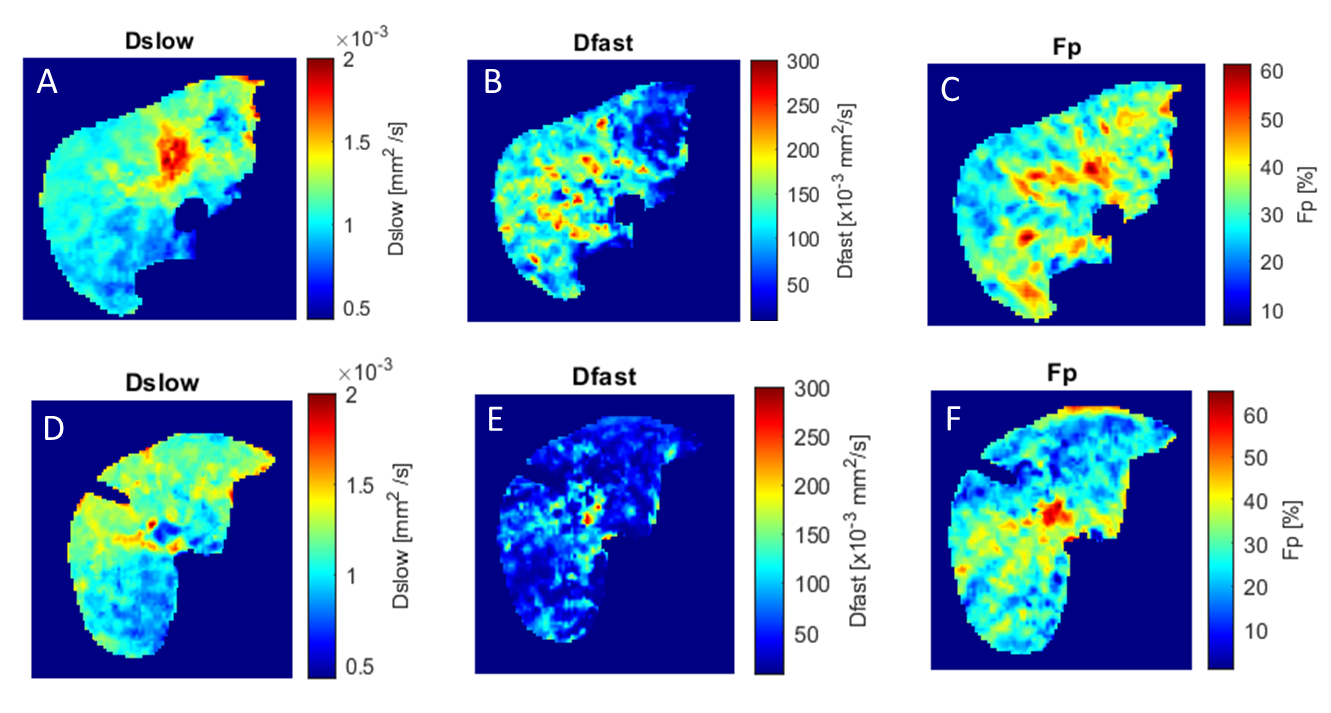
**

**Figure S1** compares IVIM parametric maps of D_slow_ (A-D), D_fast_ (B-E), and F_p_ (C-F) derived from a healthy volunteer (upper panel) and a patient with mild liver function impairments (lower panel).


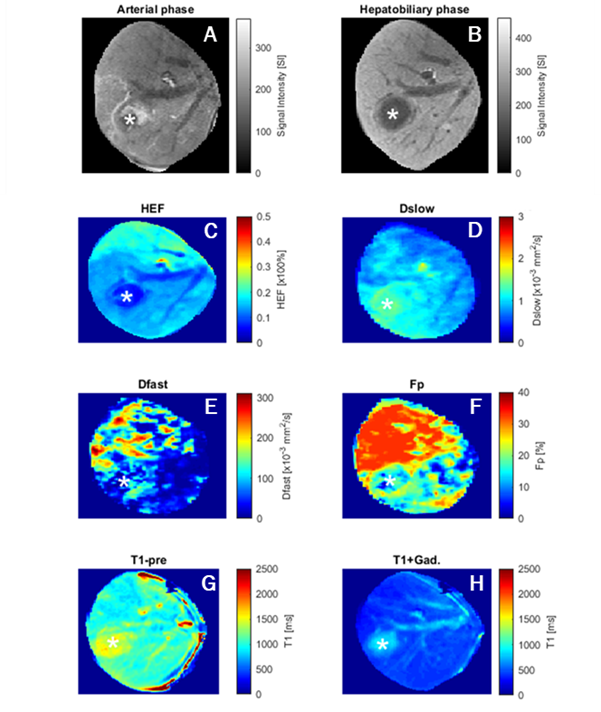


**Figure S2.** The example of segmented MRI images (arterial phase (A) and hepatobiliary phase (B)) and mp-MRI maps including HEF map (C), IVIM-derived D_slow_ (D), D_fast_ (F), and F_p_ (E), T1 mapping pre-contrast enhancement (G), and 15 min post-Gadoxetate administration (H). The asterisks indicate the tumor.
